# Dataset on electro-optically tunable smart-supercapacitors based on oxygen-excess nanograin tungsten oxide thin film

**DOI:** 10.1016/j.dib.2017.07.051

**Published:** 2017-08-01

**Authors:** Akbar I. Inamdar, Jongmin Kim, Yongcheol Jo, Hyeonseok Woo, Sangeun Cho, Sambhaji M. Pawar, Seongwoo Lee, Jayavant L. Gunjakar, Yuljae Cho, Bo Hou, Seung Nam Cha, Jungwon Kwak, Youngsin Park, Hyungsang Kim, Hyunsik Im

**Affiliations:** a Division of Physics and Semiconductor Science, Dongguk University, Seoul 04620, South Korea; b Department of Engineering Science, University of Oxford, Parks Road, OX1 3PJ, UK; c Medical Physics Department, Asan Medical Center, Seoul, South Korea; d School of Natural Science, Ulsan National Institute of Science and Technology, Ulsan 44919, Korea

**Keywords:** Multi-functional electrode, Oxygen-excess tungsten oxide, Nanograin, Electrochromism, Supercapacitor

## Abstract

The dataset presented here is related to the research article entitled “Highly Efficient Electro-optically Tunable Smart-supercapacitors Using an Oxygen-excess Nanograin Tungsten Oxide Thin Film” (Akbar et al., 2017) [9] where we have presented a nanograin WO_3_ film as a bifunctional electrode for smart supercapacitor devices. In this article we provide additional information concerning nanograin tungsten oxide thin films such as atomic force microscopy, Raman spectroscopy, and X-ray diffraction spectroscopy. Moreover, their electrochemical properties such as cyclic voltammetry, electrochemical supercapacitor properties, and electrochromic properties including coloration efficiency, optical modulation and electrochemical impedance spectroscopy are presented.

**Specifications Table**

**Table:** 

Subject area	Physics, Chemistry
More specific subject area	Materials science, Energy, Nanotechnology
Type of data	Images, Figures, Table
How data was acquired	Atomic force microscope, Raman spectroscope, X-Ray diffractometer, Electrochemical supercapacitor characteristics, Electrochromic properties, Electrochemical impedance
Data format	Raw, analyzed, etc
Experimental factors	–
Experimental features	Thin films were deposited on conducting ITO glass substrates using a RF magnetron sputtering system with different oxygen contents. Electrochemical properties of the films were investigated using potentiostat (Princeton Applied Research VersaSTAT 3) in three-electrode electrochemical cell consisting of 1 M LiClO_4_ + PC as the electrolyte.
Data source location	Division of Physics and Semiconductor Science,
	Dongguk University, Seoul 04620, South Korea
Data accessibility	Data is supplied with in this article

**Value of the data**

● The atomic force microscopy and Raman spectroscopy provide the information about morphologies such as grain size, surface roughness, and structural characteristics of the electrode films.
● X-ray diffraction spectroscopy was used to investigate the crystal structure and the phase evolution of the tungsten oxide thin films.
● Cyclic voltammetry, electrochemical charge-discharge measurements, and chronocoulometry were used to estimate specific capacitance, coloration efficiency, and optical modulation of the tungsten oxide electrode film.
● The graph of the cathodic peak current as a function of the square root of scan rate describes the information about the electrochemical reaction mechanism of the electrode.
● AC impedance spectroscopy was used to analyze the charge-discharge process of the tungsten oxide thin film.

## 1 Data

### 1.1 Thin film characterization

The dataset presented here is related to the research article entitled “Highly Efficient Electro-optically Tunable Smart-supercapacitors Using an Oxygen-excess Nanograin Tungsten Oxide Thin Film” [9]. Atomic force microscopy, Raman spectroscopy and X-ray diffraction were used to study the structural properties of the film (Figs. S1 and S2). The oxygen content in the film was varied by changing the Ar to O_2_ gas ratio: 10:0 (0% oxygen, W1 sample), 9:1 (10% oxygen, W2), 8:2 (20% oxygen, W3), 7:3 (30% oxygen, W4), and 6:4 (40% oxygen, W5). The AFM images of the electrodes are shown below. The film deposited at 20% oxygen content (W3) during sputtering shows the highest surface roughness and smallest grain size compared with the other samples. Thus, the increased roughness (or effective surface area) enhances the adsorption of Li-ion at the surface. Two characteristic Raman peaks centered at 758 and 949 cm^−1^ are associated with the O-W-O and W=O stretching mode vibrations respectively. The breadth of the O-W-O peak suggests that the film has a nanogranular structure, which is consistent with the TEM and AFM observations. Because the W3 sample shows the best electrochemical properties, we focus on this sample in the manuscript.

The XRD pattern (Fig. S2) indicates that the particle size of the obtained product might be very small which is quite obvious in sputtered tungsten oxide sample. The huge broadening of the XRD peak can be due to very tiny particles. To be more precise towards the qualitative phase analysis, we have further improved the crystallinity of the as-prepared sample by annealing it at 300 °C for 3 h. The XRD data collected on annealed sample is given separately bellow (Fig. S2). The deconvolution of the first peak observed at 2θ position 23.64° of the three different reflections 002, 020 and 200 whereas the deconvolution of second peak obtained at 33.60° is of the three different reflections 022, 20-2 and 202. The 002 reflection's 2θ position was found to be 23.12° which has corresponding inter-planar distance d~3.84 Å of monoclinic tungsten oxide symmetry.

### 1.2 Electrochemical characterization

The electrochemical characteristics of the tungsten oxide electrode were determined using cyclic voltammetry (CV), galvanostatic charge-discharge, and AC impendence analysis using a potentiostat (Princeton Applied Research VersaSTAT 3). The cathodic current measured from the CV curves between −0.9 and 0.5 V (vs. SCE) in 1 M LiClO_4_+PC electrolyte with respect to square root of scan rates 10, 20, 50, 80, and 100 mV s^−1^ is shown in Fig. S3. The linear increase of capacitive current as a function of square root of scan rate confirm the diffusion-controlled reaction based on the standard Randles–Sevcik equation.

The specific capacitance was drawn using charge-discharge measurements for all samples having different oxygen contents is presented in Fig. S4. It reveals that the sample with 20% oxygen content (W3) shows best supercapacitive performance.

Long-term cycling stability is one of important characteristics of the electrochemical supercapacitor electrodes. The electrochemical cycling stability of the W3 electrode is evaluated using CV tests for up to 2000 cycles, as shown in Fig. S5. The change in the CV area gives the information about the cycling stability of the electrode.

Subsequently the all the electrodes were tested for their electrochromic properties and their coloration efficiency (CE) and optical density (∆OD) at 630 nm is calculated using following equations presented in Fig. S6. The film are colored and bleached by applying a potential step of ±0.75 V (vs. SCE) in 1 M LiClO_4_+PC electrolyte for a fixed time of 30 s.

(1) $${\left(\Delta \mathrm{OD}\right)}_{630\;\mathrm{nm}}=\;\log\;\left(\frac{T_{b}}{T_{c}}\right)$$

(2) $${\mathrm{CE}}_{630\;\mathrm{nm}}=\;\left(\frac{{\left(\Delta \mathrm{OD}\right)}_{630\;\mathrm{nm}}}{Q/A}\right)$$

The electrochemical impedance spectroscopy of the W3 sample is elucidated after charge and discharge processes is shown in Fig. S7. The reduced impedance after the charge process corroborates the formation of metallic W^+5^ states whereas upon discharging, the metallic species becomes oxidized, increasing the impedance (Table 1).

**Table 1:** Comparison of electrochemical supercapacitor properties of various tungsten oxide thin film electrodes.

Electrode	Counter electrode	Specific capacitance	Stability (cycles)	Reference number
Oxygen-rich WO_3+δ_ nanograins	Pt coil	228 F g^−1^	2000	This work
WO_3−*x*_ nanoplates	Pt wire	20 F g^−1^	500	[1]
nanosheet-WO_3_/graphene composites	Pt foil	143.6 F g^−1^	–	[2]
Self-assembled NiWO_4_	–	173 F g^−1^	1000	[3]
WO_3_ nanorods	–	2.8 mF cm^−2^	1000	[4]
Ordered mesoporous tungsten oxide	Pt flag	199 F g^−1^	–	[5]
Mesoporous WO_3−x_/carbon nanocomposite	Pt wire	103 F g^−1^	–	[6]
Single crystalline WO_3_ nanoparticles	Pt coil	54 F g^−1^	1000	[7]
WO_3_/carbon aerogel composite	Pt coil	700 F g^−1^	1000	[7]
WO_3_/PANI composite	Pt plate	201 F g^−1^	–	[8]

## 2 Experimental design, materials and methods

For microscopies, X-ray diffraction analysis and Raman measurements, the films were grown on glass substrates using a RF-magnetron suturing technique. To characterize the tungsten oxide thin film, atomic force microscopy (AFM) in the contact mode was used. The crystal structure and the phase evolution were then studied using X-ray diffraction spectroscopy (PANalytical X'pert PRO, with Kα=1.54056 Å) and micro-Raman spectroscopy (VG Multilab 2000, Thermo VG Scientific, UK). For the electrochemical properties, the tungsten oxide thin films were grown on the conducting glass substrates. The three-electrode electrochemical cell consists of 1 M LiClO_4_ + PC as the electrolyte, a WO_3_ electrode as the working electrode, a saturated calomel electrode (SCE) as the reference electrode, and a Pt coil as the counter-electrode.
