# Supplementary material for: Dataset on electro-optically tunable smart-supercapacitors based on oxygen-excess nanograin tungsten oxide thin film
Source: Data Brief. 2017 Aug 1;14:453–7. doi: 10.1016/j.dib.2017.07.051 (PMC5552375; doi:10.1016/j.dib.2017.07.051)
Supplement: Supplementary file 1 — Supplementary material [file mmc1.doc]

**Conflict of Interest**

**Manuscript No.:** DIB-D-17-00253R1
**Title:** Dataset on Electro-optically tunable smart-supercapacitors based on oxygen-excess nanograin tungsten oxide thin film
**Journal Title:** Data in Brief

The authors whose names are listed in the above manuscript certify that they have NO affiliations with or involvement in any organization or entity with any financial interest, or non-financial interest in the subject matter or materials discussed in this manuscript.
