# Supplementary material for: Dataset on electro-optically tunable smart-supercapacitors based on oxygen-excess nanograin tungsten oxide thin film
Source: Data Brief. 2017 Aug 1;14:453–7. doi: 10.1016/j.dib.2017.07.051 (PMC5552375; doi:10.1016/j.dib.2017.07.051)
Supplement: Supplementary file 2 — Supplementary material [file mmc2.doc]

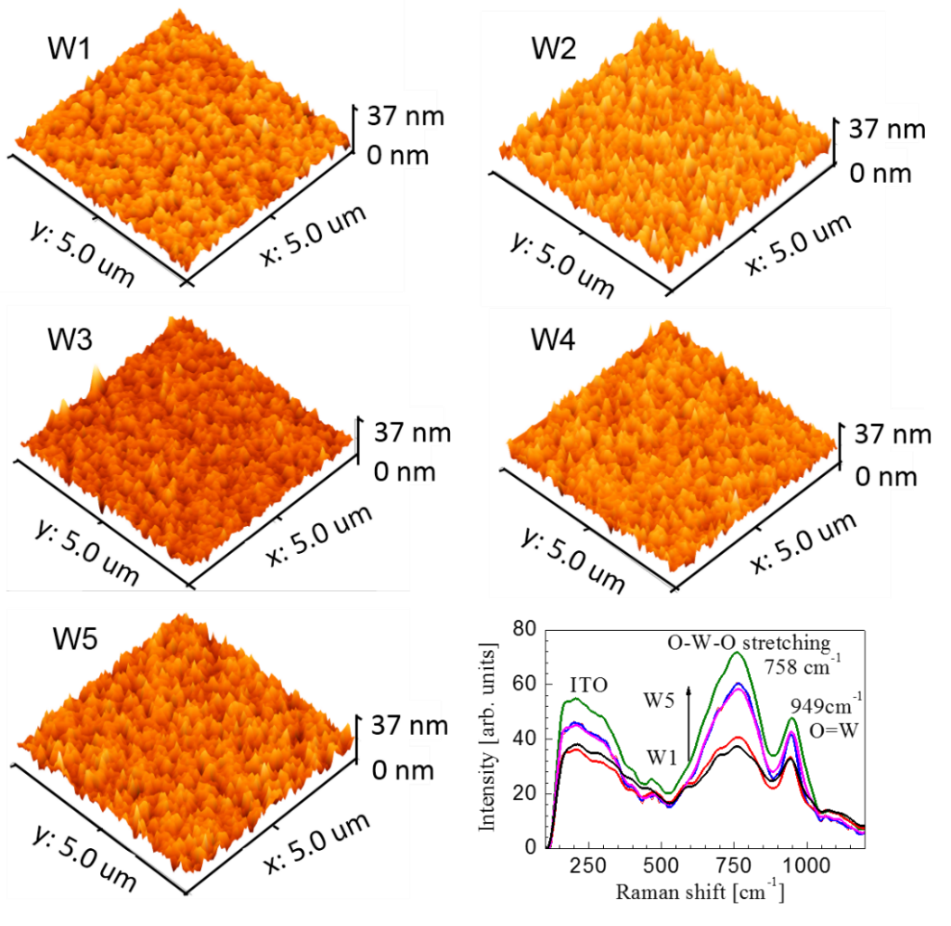
 **(a)**

 **(b)**

**Fig. S1.** (a) AFM images and Raman spectra of the WO_3_ electrodes and (b) surface roughness of the electrodes.

**Fig. S2.** X-ray diffraction pattern of the annealed sample at 300°C for 3 hours (black); The inset shows the deconvolution of two broad peaks obtained at 2θ = 23.64° and 33.60°.

**Fig. S3.** Cathodic-peak current density as a function of the square root of scan rate.

**Fig. S4.** The influence of oxygen content on the specific capacitance of tungsten oxide nanograins thin films.

**

**Fig. S5.** Long-cycled CV curves after each of 100 cycles for up to 2000 charge-discharge cycles.

**Fig. S6.** The influence of oxygen content on the coloration efficiency, optical modulation and change in the optical density (∆OD) of tungsten oxide nanograins thin films.

**Fig. S7.** (a) Nyquist plots recorded after the charge process, and (b) Nyquist plots recorded after the discharge process.
